# The Role of Oxidative Stress and Therapeutic Potential of Antioxidants in Graves’ Ophthalmopathy

**DOI:** 10.3390/biomedicines9121871

**Published:** 2021-12-10

**Authors:** Tzu-Yu Hou, Shi-Bei Wu, Hui-Chuan Kau, Chieh-Chih Tsai

**Affiliations:** 1 Department of Ophthalmology, Kaohsiung Veterans General Hospital, Kaohsiung 81362, Taiwan; houtztz@gmail.com; 2 Department of Ophthalmology, Taipei Veterans General Hospital, Taipei 11217, Taiwan; hckau1234@yahoo.com; 3 School of Medicine, National Yang Ming University, Taipei 11221, Taiwan; 4 School of Medicine, National Yang Ming Chiao Tung University, Hsinchu 30010, Taiwan; 5 Biomedical Commercialization Center, Taipei Medical University, Taipei 11031, Taiwan; barry0110@tmu.edu.tw; 6 Department of Ophthalmology, Koo Foundation Sun Yat-Sen Cancer Center, Taipei 11259, Taiwan

**Keywords:** antioxidant, Graves’ disease, Graves’ ophthalmopathy, immune, oxidative stress, reactive oxygen species

## Abstract

Graves’ ophthalmopathy (GO) is the most common extrathyroidal manifestation of Graves’ disease. It is characterized initially by an inflammatory process, followed by tissue remodeling and fibrosis, leading to proptosis, exposure keratopathy, ocular motility limitation, and compressive optic neuropathy. The pathogenic mechanism is complex and multifactorial. Accumulating evidence suggests the involvement of oxidative stress in the pathogenesis of GO. Cigarette smoking, a major risk factor for GO, has been shown to induce reactive oxygen species (ROS) generation and oxidative damage in GO orbital fibroblasts. In addition, an elevation in ROS and antioxidant enzymes is observed in tears, blood, and urine, as well as orbital fibroadipose tissues and fibroblasts from GO patients. In vitro and in vivo studies have examined the efficacy of various antioxidant supplements for GO. These findings suggest a therapeutic role of antioxidants in GO patients. This review summarizes the current understanding of oxidative stress in the pathogenesis and potential antioxidants for the treatment of GO.

## 1. Introduction

Graves’ ophthalmopathy (GO) is also known as thyroid-associated ophthalmopathy (TAO) and thyroid eye disease (TED). It is the most frequent extrathyroidal manifestation of Graves’ disease (GD). GO affects 25–50% of patients with GD [1]. It is characterized by an inflammatory process and subsequent tissue expansion, remodeling, and/or fibrosis of fibroadipose tissues and extraocular muscles in the orbit [2,3]. Although the exact pathogenesis is incompletely understood, most studies have agreed with an abnormal immune response to thyroid-stimulating hormone receptor (TSHR) and insulin-like growth factor-1 receptor (IGF-1R) in the orbit, where orbital fibroblasts appear to be the major effector cells [3,4]. Orbital fibroblasts interact with circulating T cells and respond to autoantibodies produced by B cells as well as various cytokines and perhaps as yet unrecognized stimuli. Subsequently, they proliferate and secrete glycosaminoglycans (GAGs), in particular hyaluronan, into the extracellular matrix (ECM), and differentiate into adipocytes or myofibroblasts. As a result, orbital soft tissues expand in the limited orbital space, leading to compromised orbital circulation, proptosis, lid retraction, limited ocular motility, and optic nerve compression. The mainstay treatment for moderate to severe GO is systemic glucocorticoids. However, patients with advanced GO are often unresponsive to immunosuppressive therapy and thereby require surgical intervention due to severe tissue remodeling and/or fibrosis. Clarification of the molecular mechanism of GO is critical for the development of novel treatment modalities or possible biological therapies.

Oxidative stress represents excess reactive oxygen species (ROS), which disturb the intracellular redox state, leading to peroxide and free radical accumulation and subsequent damage to proteins, lipids, and DNA in cells [5]. Common ROS include superoxide radical (O_2_^•−^), hydroxyl radical (^•^OH), hydrogen peroxide (H_2_O_2_), and lipid peroxides [6]. Biological defenses against these reactive agents include superoxide dismutase (SOD), catalase (CAT), glutathione peroxidase (GPx), and glutathione (GSH). Oxidative stress has been associated with various disorders, including neurodegenerative disease, cardiovascular disease, diabetes, and neoplasm [7,8,9,10]. Increasing studies support the involvement of oxidative stress in the pathogenesis of GD and GO. In addition, oxidative stress has been related to the release of proinflammatory cytokines, hyaluronan synthesis, as well as the proliferation and differentiation of orbital fibroblasts in GO [11]. Observation of a higher prevalence and a worse disease course of GO in smokers together with laboratory results provides evidence that ROS released from cigarette smoke stimulate GO orbital fibroblasts [12,13,14,15,16]. A number of additional in vivo and in vitro studies have confirmed the role of oxidative stress in the pathogenesis of GO [17,18,19,20,21,22,23,24,25,26,27,28,29].

In this review, we summarize the current clinical and experimental findings regarding oxidative stress in the disease process as well as the potential role of antioxidants in GO. This supports the advance in antioxidants for the treatment of GO.

## 2. Oxidative Stress in Graves’ Disease and Graves’ Ophthalmopathy

The involvement of oxidative stress in the pathogenesis of GD and GO has been examined throughout the years. GD is characterized by overproduction of thyroid hormones, leading to a hypermetabolic state with increased consumption of intracellular adenosine triphosphate (ATP) and oxygen and an accompanied increase in ROS generation [30,31,32], which in turn causes damage to thyrocytes that exacerbates autoimmune reactions as well as to extrathyroidal tissues [6]. Clinical observation in patients with GD has shown higher oxidative stress, measured by either plasma H_2_O_2_, malondialdehyde (MDA), or serum thiobarbituric acid-reacting substances (TBARSs), in subjects with hyperthyroidism than in those with euthyroidism [33,34]. Meanwhile, a correlation between the oxidative/antioxidative parameters and thyroid hormones was determined [34]. After treatment with antithyroid drugs (ATDs), the oxidative markers normalized accompanied with the restoration of euthyroidism [32,35].

Although the exact pathogenic mechanisms of GO are yet unspecified, a complex interaction between cellular and humoral immunity against autoantigens (e.g., TSHR) expressed by thyrocytes and orbital tissues as well as the following inflammatory reactions and the release of ROS in the orbit have been described [36]. Orbital fibroblasts are considered the major effector cells in the pathogenesis of GO [37]. They interact with immune cells to promote adipogenesis, hyaluronan accumulation, and soft tissue remodeling and fibrosis, particularly involving the extraocular muscles and adipose tissues in the orbit [38,39]. Bartalena et al. reported that ROS not only enhanced orbital fibroblast proliferation and GAG production but also upregulated inflammatory and other mediators, including heat shock protein 72 (HSP 72), human leukocyte antigen (HLA)-DR, and intercellular adhesion molecule-1 (ICAM-1), to promote the processes of GO [40]. In vivo and in vitro studies have confirmed ROS accompanying cigarette smoking to be related to the progression of GO [14,15,16,18]. Additionally, growing evidence supports increased levels of ROS and changes in pro/antioxidant enzymes in peripheral tissues, such as tears, blood, urine, as well as orbital fibroadipose tissues and fibroblasts of patients with GO [17,18,19,20,21,22,23,24,25,26,27,28,29]. Studies displaying the above findings are described in the following sections.

## 3. Cigarette Smoking and Graves’ Ophthalmopathy

The association of cigarette smoking with GO has long been established [41]. To date, cigarette smoking is considered the most important risk factor for the development or deterioration of GO [42]. Additionally, it is associated with a worse response to treatment for GO [12,13].

Fischli et al. found that smoking interfered with the levels of ferritin, hepcidin, as well as thyroid hormones in patients with GD, and thereby influenced the oxidative stress balance [43]. In vitro studies confirmed that cigarette smoke enhanced ROS generation and simultaneously reduced antioxidative activities [44]. Consistently, smoking stimulates ROS production in the orbital cavity, either through direct contact with the surrounding sinuses or indirectly through the systemic circulation [14]. Czarnywojtek et al. suggested the concentration of urine cotinine as a quantitative and qualitative indicator of tobacco smoking, which was found to be associated with the exacerbation of ophthalmopathy in patients with GD [45]. Yoksel et al. reported that the total oxidant status and oxidative stress index values were higher in patients with GO who smoked compared with either patients with GO who did not smoke or healthy controls [46]. Tsai et al. also found that smokers had higher urinary levels of 8-hydroxy-2′-deoxyquanosine (8-OHdG) than never smokers in patients with GO [18]. They further demonstrated that GO fibroblasts exhibited an exaggerated response to cigarette smoke extract (CSE) along with a raise in oxidative stress; fibrosis-related gene expression including apolipoprotein J, fibronectin, and connective tissue growth factor (CTGF); as well as an increase in intracellular transforming growth factor-β1 (TGF-β1) and interleukin (IL)-1β in cultured orbital fibroblasts from patients with GO [15]. These proinflammatory and fibrogenic factors could subsequently lead to orbital inflammation, fibroblast proliferation, hyaluronan accumulation, and tissue fibrosis and thereby contribute to the pathogenic processes of GO.

Smoking may cause tissue hypoxia and produce hypoxia-inducible factor-1 (HIF-1). A pronounced hypoxic response of orbital fibroblasts from patients with GO has been demonstrated [47,48]. The activation of HIF-1α and subsequent HIF-1 transcription were stimulated by smoking, in particular in patients with GO. Meanwhile, there was a correlation between HIF-1α levels and the clinical activity score (CAS). Additionally, evidence has shown an enhanced expression of growth factors, cytokines, and proinflammatory mediators, including platelet-derived growth factor (PDGF), IL-6, IL-8, and platelet-activating factor (PAF), in hypoxia [49]. Görtz et al. suggested an interaction between hypoxia and inflammation and/or immunity, which may affect tissue remodeling by stimulating angiogenesis and adipogenesis through activation of HIF-1-dependent pathways in GO orbital fibroblasts [47].

Cawood, et al. reported that CSE stimulated adipocyte differentiation in cultured orbital fibroblasts through a synergizing effect between IL-1 and ROS [16]. Yoon et al. further demonstrated that CSE induced an upregulation of ROS and adipocyte differentiation in cultured GO orbital fibroblasts and that pretreatment with quercetin or caffeine could suppress adipogenesis in cultured GO orbital fibroblasts [14,50]. The findings obtained in these studies not only provide clues for the impact of cigarette smoking on GO but also offer the possibility to treat GO with antioxidants clinically.

## 4. In Vivo Evidence of Oxidative Stress in Graves’ Ophthalmopathy

Bednarek et al. reported elevated blood levels of H_2_O_2_, lipid hydroperoxide (ROOH), TBARS, and ceruloplasmin as well as increased SOD and CAT activities but reduced GPx and glutathione reductase activities in patients with GD [24,33]. These parameters were normalized after achieving euthyroidism in the study population except those with GO, indicating that the contribution of orbital inflammation to oxidative stress was independent of thyroid hormones [33,51].

Tsai et al. detected oxidative DNA damage, which was represented by urinary 8-OHdG in patients with GO and related it to the disease activity [17,18]. They illustrated a significant positive correlation between the CAS of GO and urinary levels of 8-OhdG. Meanwhile, there was a simultaneous reduction in the disease activity and severity of GO and urinary 8-OhdG following systemic glucocorticoids treatment [17]. The effect of glucocorticoids on oxidative stress was confirmed as well. A reduction in serum MDA, which was released during lipid peroxidation, was demonstrated in patients with GO treated with glucocorticoids [25]. A subsequent study demonstrated increased concentrations of 8-OHdG and MDA in the tears of patients with GO, which was correlated with the CAS in active GO [19]. Furthermore, the impairment of thiol-disulfide homeostasis representing an antioxidant buffer system was illustrated in patients with moderate to severe GO, particularly in subjects with active disease and those who smoked [52].

## 5. In Vitro Evidence on Oxidative Stress in Graves’ Ophthalmopathy

Early in the 1990s, Heufelder et al. demonstrated an overexpression of HSP 72, which was associated with autoimmune reactions in GO fibroblasts, on H_2_O_2_ stimulation [26]. While additional treatment with either oxygen radical scavengers (i.e., diaminobenzidine, nicotinamide, and GSH) or ATDs (i.e., propylthiouracil and methimazole) diminished H_2_O_2_-induced HSP 72 expression. In 1999, Lu et al. reported an excess accumulation of GAGs induced by IL-1β in the retroocular tissues of GO, which could be partially inhibited by SOD and CAT [22]. They also demonstrated an increase in ROS as well as SOD activity in GO retroocular fibroblasts as compared with normal controls. In fact, GO orbital fibroblasts were less responsive to SOD compared with normal controls despite enhanced ROS production and concurrent SOD activation with IL-1β stimulation in orbital fibroblasts from both groups. In 1997, Burch et al. demonstrated an increase in the proliferation of GO orbital fibroblasts stimulated by O_2_^•−^ released from the xanthine oxidase/hypoxanthine system in a dose-dependent manner [27]. On the contrary, the orbital fibroblasts from the control did not respond to superoxide stimulation. The effect of ROS on orbital fibroblast proliferation could be reversed by either methimazole or antioxidants, including allopurinol and nicotinamide. These findings suggested the contribution of oxidative stress to the orbital fibroblast proliferation in patients with GO. Later, Tsai et al. described a biphasic effect of H_2_O_2_ on the viability of orbital fibroblasts selectively from patients with GO. Low levels of H_2_O_2_ enhanced the orbital fibroblast proliferation, while a concentration of H_2_O_2_ greater than 50 μM led to a cytotoxic effect. In addition, 6.25 μM H_2_O_2_ upregulated TGF-β1, IL-1β, and O_2_^•−^ in orbital fibroblasts from patients with GO [28]. It is believed that IL-1β induces inflammatory cytokines and that TGF-β1 promotes tissue remodeling and fibrosis through myofibroblast transdifferentiation in GO orbital fibroblasts [51,53]. These processes are critical in the pathogenesis of GO.

Hondur et al. investigated the oxidative stress and antioxidant activity in the orbital fibroadipose tissues, which presented with higher levels of lipid hydroperoxide as well as increased activities of SOD, glutathione reductase, and GPx in patients with moderate to severe GO compared with that in the control [20]. While the expression of GSH, representing the antioxidant level in the orbital tissues, was reduced and was negatively correlated with the clinical manifestations of patients with GO.

Marique et al. examined the extraocular muscle and adipocytes. Both of them revealed increased oxidative stress represented by 4-hydroxynonenal and concurrent upregulation of antioxidants, including peroxiredoxin 5, CAT, adiponectin, and proliferator-activated receptor gamma (PPARγ), in the specimens obtained from patients with GO as compared with the control regardless of the serum TSHR antibody levels, which, however, were related to the expression of oxidative stress and antioxidant defenses [21].

**Figure 1:**
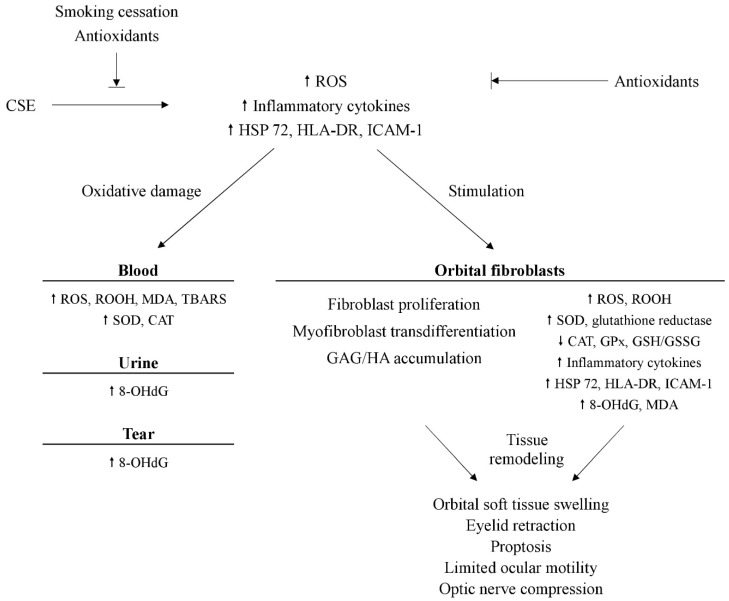
Overview of the potential mechanism of oxidative stress and possible parameters in Graves’ ophthalmopathy. OHdG: 8-hydroxy-2′-deoxyquanosine. CAT: catalase. CSE: cigarette smoke extract. GAG: glycosaminoglycans. GPx: glutathione peroxidase. GSH: glutathione. GSSG: glutathione disulfide. HA: hyaluronic acid. HLA-DR: human leukocyte antigen-DR. HSP 72: heat shock protein 72. ICAM-1: intercellular adhesion molecule-1. MDA: malondialdehyde. ROOH: lipid hydroperoxide. ROS: reactive oxygen species. SOD: superoxide dismutase. TBARS: thiobarbituric acid-reacting substances.

Tsai et al. further investigated oxidative DNA damage, lipid peroxidation, and ROS production in orbital fibroadipose tissues and cultured orbital fibroblasts [23]. The levels of 8-OHdG, MDA, O_2_^•−^, and H_2_O_2_ were higher in orbital fibroblasts from patients with GO than the control. Another study on orbital fibroblasts from the same group displayed increased manganese-dependent SOD (MnSOD) activity in addition to 8-OhdG, MDA, O_2_^•−^, and H_2_O_2_ in GO orbital fibroblasts, which, on the other hand, expressed lower GPx activity as well as the ratio between reduced GSH and glutathione disulfide (GSSG) as compared with the control [29]. Upon stimulation with H_2_O_2_, the expression of MDA, H_2_O_2_, and MnSOD activity were enhanced in GO orbital fibroblasts. Meanwhile, the CAT and GPx activities as well as the GSH/GSSG ratio were reduced. All these findings confirm the role of oxidative stress in the pathogenesis of GO and support a potential value of antioxidants in the treatment of GO (Figure 1).

## 6. Antioxidants in the Treatment of Patients with Graves’ Ophthalmopathy

The involvement of oxidative stress in the pathogenesis of GO leads to a demand for effective antioxidants to protect or treat GO. In vitro and in vivo research has described various potential antioxidants, including selenium, pentoxifylline, quercetin, enalapril, allopurinol, nicotinamide, vitamin C, N-acetylcysteine, melatonin, β-carotene, and statins. The experimental and clinical outcomes are summarized here.

### 6.1. Selenium

Selenium is a trace element naturally presenting in many foods, including Brazil nuts, tuna, shrimp, meat, egg, cereals, and other grains [54]. Evidence has shown that it contributes to normal thyroid function [55]. It incorporates selenocysteine in several selenoproteins, such as GPx, thioredoxin reductase, and iodothyronine deiodinase, representing an antioxidant to modulate oxidation–reduction reactions [56,57,58,59]. Moreover, selenium prevents NF-κB from binding to its gene promoters and consequently reduces the production of proinflammatory cytokines [60,61]. Thus, it has been regarded as a supplementary modality to treat GO [60,61].

Geographic variables are presented in selenium intake and serum concentrations [54]. It is well above recommended dietary allowance (RDA) in North America and relatively low in some European countries, especially in Eastern Europe [54]. Based on the evidence proving the impact of oxidative stress on the pathogenesis of GO, the European Group on Graves Orbitopathy (EUGOGO) carried out a randomized double-blind, placebo-controlled, multicenter clinical trial in European countries with mild selenium deficiency [62]. A total of 159 patients with mild GO were randomized to receive oral selenium (sodium selenite in a dose of 100 μg twice daily, consisting of 91.3 μg of selenium), pentoxifylline (600 mg twice daily), or placebo for 6 months. The ocular changes and quality of life were measured at 3, 6, and 12 months. At 6 months, treatment with selenium was associated with an improvement in the quality of life, overall eye assessment, and CAS, as well as a slowdown in the progression of GO, as compared with placebo. Rather, the outcome was insignificant in the pentoxifylline group. No serious adverse events were observed following selenium treatment. Therefore, the EUGOGO consensus has recommended a 6-month period of selenium supplementation in patients with mild GO of short duration [63]. However, this recommendation is not agreed with by the American Thyroid Association, as selenium deficiency is not present in the United States [64]. The risk of type 2 diabetes in patients with long-term selenium supplementation remained inconclusive [65,66,67]. Measurements of the level of serum selenium prior to selenium supplementation are advised, especially for individuals living in countries with high selenium intake, to avoid the potential disadvantages [68].

The multicenter clinical trial conducted by EUGOGO was followed by a number of in vitro studies investigating the effect of selenium on GO orbital fibroblasts. Rotondo et al. described the dual effects of selenium in GO orbital fibroblasts [69,70]. In this in vitro study, selenium, in the form of Se-methylselenocysteine (SeMCys), could suppress the proliferation of orbital fibroblasts, secretion of proinflammatory cytokines (TNF-α and IFN-γ), and release of hyaluronic acid (HA) in GO fibroblasts induced by noncytotoxic oxidative stress [69]. Whereas under cytotoxic oxidative stress, selenium could reduce cellular necrosis and apoptosis, both of which may stimulate the release of autoantigens and subsequent immune reactions [70]. One recent study further demonstrated that the production of hyaluronan and inflammatory cytokines (IL-1α, IL-8, and TNFα), as well as the generation of intracellular ROS were suppressed by selenium in cultured GO orbital fibroblasts [71]. These findings regarding the cellular mechanisms confirmed the benefits of selenium in patients with GO.

Thus, selenium supplementation is generally supported by current clinical and experimental studies in patients with mild GO, especially in those prone to selenium deficiency. Its effects on moderate-to-severe GO have not been determined and require further investigations.

### 6.2. Pentoxifylline

Pentoxifylline is a xanthine derivative primarily applied to treat peripheral vascular disease [72]. Pentoxifylline is characterized as an antioxidant that scavenges ROS and reduces inflammation [73]. In 1993, Chang et al. observed an inhibitory effect of pentoxifylline on the proliferation and GAG synthesis of fibroblasts from extraocular muscles in patients with GO [74]. Balázs et al. confirmed the immunomodulatory effect of pentoxifylline on cultured retroocular fibroblasts from patients with GO that both HLA-DR expression and GAG synthesis stimulated by cytokines including IL-1, tumor necrosis factor-α (TNF-α), and INF-γ were suppressed by pentoxifylline [75]. Pentoxifylline was further employed in euthyroid patients with moderately severe GO without corticosteroid therapy [76]. After 12 weeks of treatment consisting of initially intravenous pentoxifylline (200 mg daily for 10 days) and oral pentoxifylline (1800 mg daily for 4 weeks followed by 1200 mg daily), clinical improvement in soft tissue but not proptosis or extraocular muscle involvement, and concurrent reduction in serum levels of GAG and TNF-α were observed in 8 out of 10 patients. Finamor et al. reported a better quality of life in patients with inactive GO after six-month pentoxifylline (1200 mg daily) treatment compared to those treated with placebo [77]. In contrast, the most recent randomized, double-blind, placebo-controlled trial on the efficacy of pentoxifylline and selenium, however, did not support the benefits of pentoxifylline on the quality of life, clinical activity, or ocular signs over placebo in patients with mild GO [62]. The inconsistent results may partially be related to the participants who were in different stages (moderately severe GO, inactive GO, and mild GO, respectively) among the three studies.

### 6.3. Allopurinol and Nicotinamide

Allopurinol is indicated in hyperuricemia and gout. It shows additional benefits in hypoxic or inflammatory conditions [78]. Allopurinol elicits its antioxidant properties through enhancement of SOD activity and concurrent inhibition of xanthine oxidase, leading to a decrease in ROS [78]. Nicotinamide is a precursor of nicotinamide adenine dinucleotide (NAD), which exhibits its physiological functions in the form of NAD^+^ and nicotinamide adenine dinucleotide phosphate (NADP^+^) that involve various oxidation-reduction reactions [79]. Nicotinamide is a form of vitamin B3 occurring mainly in meat, fish, nuts, and mushrooms as well as being a nutritional supplement [80].

Allopurinol and nicotinamide could inhibit superoxide-induced Graves’ orbital fibroblast proliferation in vitro [41]. A subsequent study demonstrated that nicotinamide downregulated cytokine-stimulated HLA-DR and ICAM-1 expression in the active stage of Graves’ orbital fibroblasts [81]. Moreover, nicotinamide could suppress cellular proliferation as well as superoxide-induced HSP 72 expression in Graves’ orbital fibroblasts [40]. Regarding the clinical study on patients with mild or moderately severe, active, newly diagnosed GO, 9 (82%) of 11 patients treated with oral allopurinol (300 mg daily) and nicotinamide (300 mg daily) for three months led to a reduction in the disease severity of GO, while only 3 (27%) of 11 patients showed clinical improvement in the control group [82]. Besides, soft tissue inflammation responded more to antioxidant treatment.

### 6.4. Enalapril

Enalapril is a widely used angiotensin-converting enzyme inhibitor (ACEI) in hypertension treatment. It has been reported to protect tissues from oxidative damage by increasing antioxidant defenses [83,84]. Enalapril enhances endogenous antioxidant effects via glutathione-dependent antioxidant defense [85]. An in vitro study demonstrated the antiproliferative and hyaluronan-suppressing effects in orbital fibroblasts from individuals with/without GO [86]. One clinical study evaluated 12 patients with mild GO treated with enalapril (5 mg daily) for 6 months [87]. Clinical improvements, including exophthalmos, CAS, lid retraction, and quality of life, were demonstrated [87]. No significant side effects of enalapril, such as dizziness, hypotension, and hyperkalemia, were observed. The benefit of enalapril on GO might be associated with various effects, including the antioxidant activity, inhibition of orbital fibroblast proliferation and HA deposition, downregulation of adipogenesis, and prevention of TGF-β synthesis [87].

To date, only the antioxidants mentioned above (i.e., selenium, pentoxifylline, allopurinol and nicotinamide, and enalapril) have been clinically utilized in patients with GO (Table 1). Most clinical studies reveal that antioxidants may be beneficial for those patients with mild or mild to moderate GO [62,76,77,82,87]. It is possible that early treatment of mild GO with antioxidants may reduce the risk of progression to a more severe form of GO in which a therapeutic challenge often remains. Further large randomized controlled clinical studies are required to confirm these issues.

### 6.5. Quercetin

Quercetin is a polyphenolic flavonoid compound. It is one of the most abundant flavonols widely distributed in fruits, vegetables, red wine, and tea [88]. It has attracted more attention for medicinal applications in recent years. It participates in a number of biological actions, including antioxidation, anti-inflammation, antiproliferation, and modulation of various protein and lipid kinase pathways [89]. Actually, its antioxidant and anti-inflammatory effect might contribute to the treatment of GO and other ophthalmic diseases [88].

Quercetin is widely utilized in botanical medicine and traditional Chinese medicine as it is a potent antioxidant [90]. The antioxidant activity of quercetin is achieved by influencing glutathione, enzymes, signal transduction pathways, and ROS production. Particularly when it forms complexes with metal ions, its bioavailability and antioxidant effect are enhanced [90]. Yoon et al. investigated the effect of quercetin in primary cultured GO orbital fibroblasts and reported that quercetin could reduce ROS generation and adipocyte differentiation induced by CSE and H_2_O_2_ [14]. In addition, it has been suggested that quercetin negatively regulates fibroblast proliferation, inflammation, hyaluronan production, adipogenesis, and fibrogenesis in the primary cultures of GO orbital fibroblasts [91,92,93,94]. The clinical efficacy of quercetin in managing GO is worth further investigation.

### 6.6. Vitamin C, N-Acetylcysteine, and Melatonin

Vitamin C or ascorbic acid is an essential nutrient involved in numerous biological activities that helps wound healing and collagen synthesis. It is also a reductant that donates electrons in redox chemical reactions [95]. N-acetylcysteine is a precursor of glutathione that reduces free radicals physiologically [96]. Melatonin is known to be released by the pineal gland to control the sleep–wake cycle. It has been found to be a free radical scavenger and upregulate antioxidants like SOD, GPx, glutathione reductase, and CAT [97,98].

In the in vitro study conducted by Tsai et al., N-acetylcysteine and vitamin C reversed fibroblast proliferation and the expression of TGF-β1, IL-1β, and superoxide anion in Graves’ orbital fibroblasts induced by H_2_O_2_ [42]. Another in vitro study performed in primary cultures of orbital fibroblasts illustrated that vitamin C, N-acetylcysteine, and melatonin reduced glutathione disulfide (GSSG) release stimulated by H_2_O_2_ [99]. In addition, fibroblast proliferation could be inhibited by vitamin C and N-acetylcysteine, while IFN-γ release could be reduced by N-acetylcysteine and melatonin selectively in Graves’ orbital fibroblasts. HA and IL1-β synthesis, on the other hand, could be reduced by N-acetylcysteine and melatonin in orbital fibroblasts from either patients with GO or normal controls. The therapeutic effects of these antioxidant agents in clinical practice need advanced investigation.

### 6.7. β-Carotene

β-carotene is a carotenoid, which is the primary dietary provitamin A. It contributes to the red-orange color in various fruits and vegetables [100]. It is believed to be a radical-trapping antioxidant [101]. An experimental study confirmed the features of antioxidation anti-inflammation, and antiproliferation of β-carotene, which reduced H_2_O_2_-induced GSSG, a parameter of oxidative stress, and IL-1β production as well as cell proliferation in cultured orbital fibroblasts from patients with GO [102]. The therapeutic role of β-carotene in GO needs further investigation.

### 6.8. Statins

Statins are inhibitors of 3-hydroxy-3-methylglutaryl coenzyme A (HMG-CoA) reductase and are widely used to treat hypercholesterolemia [103]. Aside from the effect on cholesterol biosynthesis, they have been found to reduce oxidative stress through a variety of mechanisms, including a reduction of plasma lipids, radical scavenging, and inhibition of vascular NAD(P)H oxidase [103]. In recent years, statins have been shown to reduce the risks of GO in patients with GD [104,105]. One randomized clinical trial confirmed the benefit of additional oral atorvastatin to intravenous glucocorticoids on the clinical outcomes and quality of life in patients with moderate-to-severe active GO who had hypercholesterolemia [106]. The mechanisms underlying the potential link between GO and cholesterol may reflect the increased load of free fatty acids. In hepatocytes, the increased load of free fatty acids is followed by an altered function of mitochondria and endoplasmic reticulum, which ultimately causes the release of ROS and proinflammatory cytokines, which are both involved in the pathogenesis of GO [107].

Although antioxidants may not replace glucocorticoids as the first-line treatment for GO, they should be considered adjunctive therapies. The 2021 EUGOGO clinical practice guidelines recommended patients with mild and active GO of recent onset to receive 6-month selenium supplementation to improve ocular manifestations and quality of life as well as to prevent GO progression [108]. In addition, several antioxidants, such as curcumin and quercetin, provide various physiological functions, including anti-inflammation, antioxidation, antiproliferation, and antifibrosis, that could modulate diverse processes for GO [89,109,110].

## 7. Conclusions

This review summarizes the current understanding of oxidative stress in the pathogenesis of GO and potential antioxidants for the treatment of GO. The involvement of oxidative stress in the pathogenesis of GO could be supported by a worse disease course in smokers as well as enhanced fibrogenesis and adipogenesis in GO orbital tissues treated with CSE. An elevation of peripheral oxidative markers was observed in patients with either GD or GO. Examination of the orbital tissues from patients with GO also demonstrated the upregulation of ROS as well as excess cell proliferation and GAG synthesis, which could be reversed by antioxidative agents and ATDs. The activities of free radical scavengers in the GO orbital fibroblasts or fibroadipose tissues were, however, inconclusive. The effectiveness of antioxidant supplements, including selenium, pentoxifylline, quercetin, enalapril, allopurinol, nicotinamide, vitamin C, N-acetylcysteine, melatonin, β-carotene, and statins, have been evaluated. The efficacy in the improvement of clinical manifestations and/or inhibition of disease progression in mild GO is relatively evident in selenium. Increasing evidence of the role of oxidative stress in GO encourages more investigations into new potential antioxidants in the treatment of GO. Especially, early treatment of mild GO may effectively limit the risk of progression to a more severe form of GO, which often remains a therapeutic challenge.

## Figures and Tables

**Table 1:** Clinical studies on the efficacy of antioxidants in Graves’ ophthalmopathy.

Antioxidant	Study Design	Dosage and Interval	Disease Status	Case Number	Placebo	Clinical Outcome	Adverse Event
Selenium	RCT [62]	Oral sodium selenite (200 μg/day)/selenium (91.3 μg/day) for 6 months	Mild GO	54	+	Improved QOL, CAS, and ocular signs; slowdown of GO progression	-
Pentoxifylline	Observational study [76]	IV 200 mg/day for 10 days, followed by oral 1800 mg/day for 4 weeks, and then oral 1200 mg/day (overall 12 weeks)	Moderate GO	10	−	Improved soft tissue inflammation; no benefits on proptosis and extraocular muscle involvement	N/A *
Pentoxifylline	RCT [77]	Oral 1200 mg/day for 6 months	Inactive GO	9	+	Improved QOL and proptosis	Minor gastrointestinal side effects
Pentoxifylline	RCT [62]	Oral 1200 mg/day for 6 months	Mild GO	48	+	No benefits on QOL, CAS, or ocular signs	Skin and gastrointestinal disorders
Allopurinol and nicotinamide	Nonrandomized comparative study [82]	Oral allopurinol (300 mg/day) and nicotinamide (300 mg/day) for 3 months	Mild or moderate GO	11	+	Improved symptoms and ocular signs, especially soft tissue inflammation	-
Enalapril	Observational study [87]	Oral 5 mg/day for 6 months	Mild GO	12	−	Improved QOL, CAS, exophthalmos, and lid retraction	-

CAS: clinical activity score. GO: Graves’ ophthalmopathy. IV: intravenous. QOL: quality of life. RCT: randomized controlled trial. *: N/A indicates that the data was not available or the study did not assess the gene of interest.

## Data Availability

Not applicable.

## References

[1] Garrity J.A., Bahn R.S. (2006). Pathogenesis of graves ophthalmopathy: Implications for prediction, prevention, and treatment. Am. J. Ophthalmol..

[2] Pouso-Diz J.M., Abalo-Lojo J.M., Gonzalez F. (2020). Thyroid eye disease: Current and potential medical management. Int. Ophthalmol..

[3] Khong J.J., McNab A.A., Ebeling P.R., Craig J.E., Selva D. (2016). Pathogenesis of thyroid eye disease: Review and update on molecular mechanisms. Br. J. Ophthalmol..

[4] Dik W.A., Virakul S., van Steensel L. (2016). Current perspectives on the role of orbital fibroblasts in the pathogenesis of Graves’ ophthalmopathy. Exp. Eye Res..

[5] Birnboin H.C. (1986). DNA strand breaks in human leukocytes induced by super-oxide anion, hydrogen peroxide and tumor promoters are repaired slowly compared to breaks induced by ionizing radiation. Carcinogenesis.

[6] Lanzolla G., Marcocci C., Marinò M. (2020). Oxidative stress in Graves disease and Graves orbitopathy. Eur. Thyroid J..

[7] Chen Z., Zhong C. (2014). Oxidative stress in Alzheimer’s disease. Neurosci. Bull..

[8] Kattoor A.J., Pothineni N.V.K., Palagiri D., Mehta J.L. (2017). Oxidative stress in atherosclerosis. Curr. Atheroscler. Rep..

[9] Senoner T., Dichtl W. (2019). Oxidative stress in cardiovascular diseases: Still a therapeutic target?. Nutrients.

[10] Gorrini C., Harris I.S., Mak T.W. (2013). Modulation of oxidative stress as an anticancer strategy. Nat. Rev. Drug. Discov..

[11] Marcocci C., Leo M., Altea M.A. (2012). Oxidative stress in graves’ disease. Eur. Thyroid. J..

[12] Bartalena L., Marcocci C., Tanda M.L., Manetti L., Dell’Unto E., Bartolomei M.P., Nardi M., Martino E., Pinchera A. (1998). Cigarette smoking and treatment outcomes in Graves ophthalmopathy. Ann. Intern. Med..

[13] Eckstein A., Quadbeck B., Mueller G., Rettenmeier A.W. (2003). Hoermann, R.; Mann, K.; Steuhl, P.; Esser, J. Impact of smoking on the response to treatment of thyroid associated ophthalmopathy. Br. J. Ophthalmol..

[14] Yoon J.S., Lee H.J., Chae M.K., Lee S.Y., Lee E.J. (2013). Cigarette smoke extract-induced adipogenesis in Graves’ orbital fibroblasts is inhibited by quercetin via reduction in oxidative stress. J. Endocrinol..

[15] Kau H.C., Wu S.B., Tsai C.C., Liu C.J., Wei Y.H. (2016). Cigarette smoke extract-induced oxidative stress and fibrosis-related genes expression in orbital fibroblasts from patients with Graves’ ophthalmopathy. Oxid. Med. Cell. Longev..

[16] Cawood T.J., Moriarty P., O’Farrelly C., O’Shea D. (2007). Smoking and thyroid-associated ophthalmopathy: A novel explanation of the biological link. J. Clin. Endocrinol. Metab..

[17] Tsai C.C., Kao S.C., Cheng C.Y., Kau H.C., Hsu W.M., Lee C.F., Wei Y.H. (2007). Oxidative stress change by systemic corticosteroid treatment among patients having active graves ophthalmopathy. Arch. Ophthalmol..

[18] Tsai C.C., Cheng C.Y., Liu C.Y., Kao S.C., Kau H.C., Hsu W.M., Wei Y.H. (2009). Oxidative stress in patients with Graves’ ophthalmopathy: Relationship between oxidative DNA damage and clinical evolution. Eye.

[19] Choi W., Li Y., Ji Y.S., Yoon K.C. (2018). Oxidative stress markers in tears of patients with Graves’ orbitopathy and their correlation with clinical activity score. BMC Ophthalmol..

[20] Hondur A., Konuk O., Dincel A.S., Bilgihan A., Unal M., Hasanreisoglu B. (2008). Oxidative stress and antioxidant activity in orbital fibroadipose tissue in Graves’ ophthalmopathy. Curr. Eye Res..

[21] Marique L., Senou M., Craps J., Delaigle A., Van Regemorter E., Wérion A., Van Regemorter V., Mourad M., Nyssen-Behets C., Lengelé B. (2015). Oxidative stress and upregulation of antioxidant proteins, including adiponectin, in extraocular muscular cells, orbital adipocytes, and thyrocytes in Graves’ disease associated with orbitopathy. Thyroid.

[22] Lu R., Wang P., Wartofsky L., Sutton B.D., Zweier J.L., Bahn R.S., Garrity J., Burman K.D. (1999). Oxygen free radicals in interleukin-1beta-induced glycosaminoglycan production by retro-ocular fibroblasts from normal subjects and Graves’ ophthalmopathy patients. Thyroid.

[23] Tsai C.C., Wu S.B., Cheng C.Y., Kao S.C., Kau H.C., Chiou S.H., Hsu W.M., Wei Y.H. (2010). Increased oxidative DNA damage, lipid peroxidation, and reactive oxygen species in cultured orbital fibroblasts from patients with Graves’ ophthalmopathy: Evidence that oxidative stress has a role in this disorder. Eye.

[24] Bednarek J., Wysocki H., Sowinski J. (2004). Peripheral parameters of oxidative stress in patients with infiltrative Graves’ ophthalmopathy treated with corticosteroids. Immunol. Lett..

[25] Akarsu E., Buyukhatipoglu H., Aktaran S., Kurtul N. (2011). Effects of pulse methylprednisolone and oral methylprednisolone treatments on serum levels of oxidative stress markers in Graves’ ophthalmopathy. Clin. Endocrinol..

[26] Heufelder A.E., Wenzel B.E., Bahn R.S. (1992). Methimazole and propylthiouracil inhibit the oxygen free radical-induced expression of a 72 kilodalton heat shock protein in Graves’ retroocular fibroblasts. J. Clin. Endocrinol. Metab..

[27] Burch H.B., Lahiri S., Bahn R.S., Barnes S. (1997). Superoxide radical production stimulates retroocular fibroblast proliferation in Graves’ ophthalmopathy. Exp. Eye Res..

[28] Tsai C.C., Wu S.B., Kao S.C., Kau H.C., Lee F.L., Wei Y.H. (2013). The protective effect of antioxidants on orbital fibroblasts from patients with Graves’ ophthalmopathy in response to oxidative stress. Mol. Vis..

[29] Tsai C.C., Wu S.B., Cheng C.Y., Kao S.C., Kau H.C., Lee S.M., Wei Y.H. (2011). Increased response to oxidative stress challenge in Graves’ ophthalmopathy orbital fibroblasts. Mol. Vis..

[30] Asayama K., Kato K. (1990). Oxidative muscular injury and its relevance to hyperthyroidism. Free Radic. Biol. Med..

[31] Venditti P., Balestrieri M., Di Meo S., De Leo T. (1997). Effect of thyroid state on lipid peroxidation, antioxidant defences, and susceptibility to oxidative stress in rat tissues. J. Endocrinol..

[32] Abalovich M., Llesuy S., Gutierrez S., Repetto M. (2003). Peripheral parameters of oxidative stress in Graves’ disease: The effects of methimazole and 131 iodine treatments. Clin. Endocrinol..

[33] Bednarek J., Wysocki H., Sowinski J. (2005). Oxidative stress peripheral parameters in Graves’ disease: The effect of methimazole treatment in patients with and without infiltrative ophthalmopathy. Clin. Biochem..

[34] Aslan M., Cosar N., Celik H., Aksoy N., Dulger A.C., Begenik H., Soyoral Y.U., Kucukoglu M.E., Selek S. (2011). Evaluation of oxidative status in patients with hyperthyroidism. Endocrine.

[35] Cetinkaya A., Kurutas E.B., Buyukbese M.A., Kantarceken B., Bulbuloglu E. (2005). Levels of malondialdehyde and superoxide dismutase in subclinical hyperthyroidism. Mediators Inflamm..

[36] Smith T.J., Hegedüs L. (2016). Graves’ Disease. N. Engl. J. Med..

[37] Smith T.J., Tsai C.C., Shih M.J., Tsui S., Chen B., Han R., Naik V., King C.S., Press C., Kamat S. (2008). Unique attributes of orbital fibroblasts and global alterations in IGF-1 receptor signaling could explain thyroid-associated ophthalmopathy. Thyroid.

[38] Marinò M., Rotondo Dottore G., Ionni I., Lanzolla G., Sabini E., Ricci D., Sframeli A., Mazzi B., Menconi F., Latrofa F. (2019). Serum antibodies against the insulin-like growth factor-1 receptor (IGF-1R) in Graves’ disease and Graves’ orbitopathy. J. Endocrinol. Investig..

[39] Bartalena L., Fatourechi V. (2014). Extrathyroidal manifestations of Graves’ disease: A 2014 update. J. Endocrinol. Investig..

[40] Bartalena L., Tanda M.L., Piantanida E., Lai A. (2003). Oxidative stress and Graves’ ophthalmopathy: In vitro studies and therapeutic implications. Biofactors.

[41] Bartalena L., Martino E., Marcocci C., Bogazzi F., Panicucci M., Velluzzi F., Loviselli A., Pinchera A. (1989). More on smoking habits and Graves’ ophthalmopathy. J. Endocrinol. Investig..

[42] Stan M.N., Bahn R.S. (2010). Risk factors for development or deterioration of Graves’ ophthalmopathy. Thyroid.

[43] Fischli S., von V., Trummler M., Konrad D., Wueest S., Ruefer A., Heering K., Streuli R., Steuer C., Bernasconi L. (2017). Iron metabolism in patients with Graves’ hyperthyroidism. Clin. Endocrinol..

[44] Wiersinga W.M. (2013). Smoking and thyroid. Clin. Endocrinol..

[45] Czarnywojtek A., Zgorzalewicz-Stachowiak M., Florek E., Piekoszewski W., Warmuz-Stangierska I., Kulińska-Niedziela I., Komar-Rychlicka K., Sowiński J. (2006). The level of cotinine-marker of tobacco smoking, in patients with hyperthyroidism. Endokrynol. Pol..

[46] Yuksel N., Yaman D., Tugce Pasaoglu O., Pasaoglu H. (2020). The Effect of Smoking on Mitochondrial Biogenesis in Patients with Graves Ophthalmopathy. Ophthalmic. Plast. Reconstr. Surg..

[47] Görtz G.E., Horstmann M., Aniol B., Reyes B.D., Fandrey J., Eckstein A., Berchner-Pfannschmidt U. (2016). Hypoxia-Dependent HIF-1 Activation Impacts on Tissue Remodeling in Graves’ Ophthalmopathy-Implications for Smoking. J. Clin. Endocrinol. Metab..

[48] Malkov M.I., Lee C.T., Taylor C.T. (2021). Regulation of the Hypoxia-Inducible Factor (HIF) by Pro-Inflammatory Cytokines. Cells.

[49] Tamm M., Bihl M., Eickelberg O., Stulz P., Perruchoud A.P., Roth M. (1998). Hypoxia-induced interleukin-6 and interleukin-8 production is mediated by platelet-activating factor and platelet-derived growth factor in primary human lung cells. Am. J. Respir. Cell Mol. Biol..

[50] Ko J., Kim J.Y., Kim J.W., Yoon J.S. (2020). Anti-oxidative and anti-adipogenic effects of caffeine in an in vitro model of Graves’ orbitopathy. Endocr. J..

[51] Londzin-Olesik M., Kos-Kudła B., Nowak A., Wielkoszyński T., Nowak M. (2020). The effect of thyroid hormone status on selected antioxidant parameters in patients with Graves’ disease and active thyroid-associated orbitopathy. Endokrynol. Pol..

[52] Yuksel N., Tanriverdi B., Ipteç B., Erel O. (2019). Thiol-disulfide homeostasis as an oxidative stress marker in patients with Graves’ ophthalmopathy. Orbit.

[53] Li H., Ma C., Liu W., He J., Li K. (2020). Gypenosides Protect Orbital Fibroblasts in Graves Ophthalmopathy via Anti-Inflammation and Anti-Fibrosis Effects. Invest. Ophthalmol. Vis. Sci..

[54] Rayman M.P. (2000). The importance of selenium to human health. Lancet.

[55] Köhrle J. (2015). Selenium and the thyroid. Curr. Opin. Endocrinol. Diabetes. Obes..

[56] Steinbrenner H., Speckmann B., Klotz L.O. (2016). Selenoproteins: Antioxidant selenoenzymes and beyond. Arch. Biochem. Biophys..

[57] Wrobel J.K., Power R., Toborek M. (2016). Biological activity of selenium: Revisited. IUBMB Life.

[58] Burk R.F., Hill K.E. (2015). Regulation of selenium metabolism and transport. Annu. Rev. Nutr..

[59] Marinò M., Dottore G.R., Leo M., Marcocci C. (2018). Mechanistic Pathways of Selenium in the Treatment of Graves’ Disease and Graves’ Orbitopathy. Horm. Metab. Res..

[60] Duntas L.H. (2012). The evolving role of selenium in the treatment of graves’ disease and ophthalmopathy. J. Thyroid. Res..

[61] Duntas L.H., Benvenga S. (2015). Selenium: An Element for Life. Endocrine.

[62] Marcocci C., Kahaly G.J., Krassas G.E., Bartalena L., Prummel M., Stahl M., Altea M.A., Nardi M., Pitz S., Boboridis K. (2011). Selenium and the course of mild Graves’ orbitopathy. N. Engl. J. Med..

[63] Bartalena L., Baldeschi L., Boboridis K., Eckstein A., Kahaly G.J., Marcocci C., Perros P., Salvi M., Wiersinga W.M., European Group on Graves’ Orbitopathy (EUGOGO) (2016). The 2016 European Thyroid Association/European Group on Graves’ Orbitopathy Guidelines for the Management of Graves’ Orbitopathy. Eur. Thyroid J..

[64] Bednarczuk T., Schomburg L. (2020). Challenges and perspectives of selenium supplementation in Graves’ disease and orbitopathy. Hormones.

[65] Wei J., Zeng C., Gong Q.Y., Yang H.B., Li X.X., Lei G.H., Yang T.B. (2015). The association between dietary selenium intake and diabetes: A cross-sectional study among middle-aged and older adults. Nutr. J..

[66] Karalis D.T. (2019). The Beneficiary Role of Selenium in Type II Diabetes: A Longitudinal Study. Cureus.

[67] Rayman M.P., Blundell-Pound G., Pastor-Barriuso R., Guallar E., Steinbrenner H., Stranges S. (2012). A randomized trial of selenium supplementation and risk of type-2 diabetes, as assessed by plasma adiponectin. PLoS ONE.

[68] Lanzolla G., Marinò M., Marcocci C. (2021). Selenium in the Treatment of Graves’ Hyperthyroidism and Eye Disease. Front. Endocrinol..

[69] Rotondo Dottore G., Leo M., Casini G., Latrofa F., Cestari L., Sellari-Franceschini S., Nardi M., Vitti P., Marcocci C., Marinò M. (2017). Antioxidant actions of selenium in orbital fibroblasts: A basis for the effects of selenium in Graves’ orbitopathy. Thyroid.

[70] Rotondo Dottore G., Chiarini R., De Gregorio M., Leo M., Casini G., Cestari L., Sellari-Franceschini S., Nardi M., Vitti P., Marcocci C. (2017). Selenium rescues orbital fibroblasts from cell death induced by hydrogen peroxide: Another molecular basis for the effects of selenium in Graves’ orbitopathy. Endocrine.

[71] Kim B.Y., Jang S.Y., Choi D.H., Jung C.H., Mok J.O., Kim C.H. (2021). Anti-inflammatory and Antioxidant Effects of Selenium on Orbital Fibroblasts of Patients With Graves Ophthalmopathy. Ophthalmic Plast. Reconstr. Surg..

[72] Broderick C., Forster R., Abdel M., Salhiyyah K. (2020). Pentoxifylline for intermittent claudication. Cochrane. Database. Syst. Rev..

[73] Bhat V.B., Madyastha K.M. (2001). Antioxidant and radical scavenging properties of 8-oxo derivatives of xanthine drugs pentoxifylline and lisofylline. Biochem. Biophys. Res. Commun..

[74] Chang C.C., Chang T.C., Kao S.C., Kuo Y.F., Chien L.F. (1993). Pentoxifylline inhibits the proliferation and glycosaminoglycan synthesis of cultured fibroblasts derived from patients with Graves’ ophthalmopathy and pretibial myxoedema. Acta. Endocrinol..

[75] Balázs C., Kiss E., Farid N.R. (1997). Inhibitory effect of pentoxifylline on HLA-DR expression and glycosaminoglycan synthesis of retrobulbar fibroblasts induced by interferon gamma. Acta. Microbiol. Immunol. Hung..

[76] Balazs C., Kiss E., Vamos A., Molnar I., Farid N.R. (1997). Beneficial effect of pentoxifylline on thyroid associated ophthalmopathy (TAO) *: A pilot study. J. Clin. Endocrinol. Metab..

[77] Finamor F.E., Martins J.R., Nakanami D., Paiva E.R., Manso P.G., Furlanetto R.P. (2004). Pentoxifylline (PTX): An alternative treatment in Graves ophthalmopathy (inactive phase): Assessment by a disease specific quality of life questionnaire and by exophthalmometry in a prospective randomized trial. Eur. J. Ophthalmol..

[78] Zajączkowski S., Ziółkowski W., Badtke P., Zajączkowski M.A., Flis D.J., Figarski A., Smolińska-Bylańska M., Wierzba T.H. (2018). Promising effects of xanthine oxidase inhibition by allopurinol on autonomic heart regulation estimated by heart rate variability (HRV) analysis in rats exposed to hypoxia and hyperoxia. PLoS ONE.

[79] Belenky P., Bogan K.L., Brenner C. (2007). NAD+ metabolism in health and disease. Trends. Biochem. Sci..

[80] Bender D.A. (2003). Nutritional Biochemistry of the Vitamins.

[81] Hiromatsu Y., Yang D., Miyake I., Koga M., Kameo J., Sato M., Inoue Y., Nonaka K. (1998). Nicotinamide decreases cytokine-induced activation of orbital fibroblasts from patients with thyroid-associated ophthalmopathy. J. Clin. Endocrinol. Metab..

[82] Bouzas E.A., Karadimas P., Mastorakos G., Koutras D.A. (2000). Antioxidant agents in the treatment of Graves’ ophthalmopathy. Am. J. Ophthalmol..

[83] de Cavanagh E.M., Fraga C.G., Ferder L., Inserra F. (1997). Enalapril and captopril enhance antioxidant defenses in mouse tissues. Am. J. Physiol..

[84] Chandran G., Sirajudeen K.N., Yusoff N.S., Swamy M., Samarendra M.S. (2014). Effect of the antihypertensive drug enalapril on oxidative stress markers and antioxidant enzymes in kidney of spontaneously hypertensive rat. Oxid. Med. Cell. Longev..

[85] de Cavanagh E.M., Inserra F., Ferder L., Fraga C.G. (2000). Enalapril and captopril enhance glutathione-dependent antioxidant defenses in mouse tissues. Am. J. Physiol. Regul. Integr. Comp. Physiol..

[86] Botta R., Lisi S., Marcocci C., Sellari-Franceschini S., Rocchi R., Latrofa F., Menconi F., Altea M.A., Leo M., Sisti E. (2013). Enalapril reduces proliferation and hyaluronic acid release in orbital fibroblasts. Thyroid.

[87] Ataabadi G., Dabbaghmanesh M.H., Owji N., Bakhshayeshkaram M., Montazeri-Najafabady N. (2020). Clinical Features of Graves’ Ophthalmopathy and Impact of Enalapril on the Course of Mild Graves’ Ophthalmopathy: A Pilot Study. Endocr. Metab. Immune. Disord. Drug. Targets.

[88] Zhao L., Wang H., Du X. (2021). The therapeutic use of quercetin in ophthalmology: Recent applications. Biomed. Pharmacother..

[89] Williams R.J., Spencer J.P., Rice-Evans C. (2004). Flavonoids: Antioxidants or signalling molecules?. Free Radic. Biol. Med..

[90] Xu D., Hu M.J., Wang Y.Q., Cui Y.L. (2019). Antioxidant Activities of Quercetin and Its Complexes for Medicinal Application. Molecules.

[91] Lisi S., Botta R., Lemmi M., Sellari-Franceschini S., Altea M.A., Sisti E., Casini G., Nardi M., Marcocci C., Pinchera A. (2011). Quercetin decreases proliferation of orbital fibroblasts and their release of hyaluronic acid. J. Endocrinol. Investig..

[92] Yoon J.S., Lee H.J., Choi S.H., Chang E.J., Lee S.Y., Lee E.J. (2011). Quercetin inhibits IL-1β-induced inflammation, hyaluronan production and adipogenesis in orbital fibroblasts from Graves’ orbitopathy. PLoS ONE.

[93] Yoon J.S., Chae M.K., Lee S.Y., Lee E.J. (2012). Anti-inflammatory effect of quercetin in a whole orbital tissue culture of Graves’ orbitopathy. Br. J. Ophthalmol..

[94] Yoon J.S., Chae M.K., Jang S.Y., Lee S.Y., Lee E.J. (2012). Antifibrotic effects of quercetin in primary orbital fibroblasts and orbital fat tissue cultures of Graves’ orbitopathy. Investig. Ophthalmol. Vis. Sci..

[95] Padayatty S.J., Katz A., Wang Y., Eck P., Kwon O., Lee J.H., Chen S., Corpe C., Dutta A., Dutta S.K. (2003). Vitamin C as an antioxidant: Evaluation of its role in disease prevention. J. Am. Coll. Nutr..

[96] Zafarullah M., Li W.Q., Sylvester J., Ahmad M. (2003). Molecular mechanisms of N-acetyl-l-cysteine actions. Cell. Mol. Life. Sci..

[97] Sharafati-Chaleshtori R., Shirzad H., Rafieian-Kopaei M., Soltani A. (2017). Melatonin and human mitochondrial diseases. J. Res. Med. Sci..

[98] Jockers R., Delagrange P., Dubocovich M.L., Markus R.P., Renault N., Tosini G., Cecon E., Zlotos D.P. (2016). Update on melatonin receptors: IUPHAR Review 20. Br. J. Pharmacol..

[99] Rotondo Dottore G., Ionni I., Menconi F., Casini G., Sellari-Franceschini S., Nardi M., Vitti P., Marcocci C., Marinò M. (2018). Action of three bioavailable antioxidants in orbital fibroblasts from patients with Graves’ orbitopathy (GO): A new frontier for GO treatment?. J. Endocrinol. Investig..

[100] van Bennekum A., Werder M., Thuahnai S.T., Han C.H., Duong P., Williams D.L., Wettstein P., Schulthess G., Phillips M.C., Hauser H. (2005). Class B scavenger receptor-mediated intestinal absorption of dietary beta-carotene and cholesterol. Biochemistry.

[101] Fazal Y., Fatima S.N., Shahid S.M., Mahboob T. (2016). Nephroprotective effects of b-carotene on ACE gene expression, oxidative stress and antioxidant status in thioacetamide induced renal toxicity in rats. Pak. J. Pharm. Sci..

[102] Rotondo Dottore G., Ionni I., Menconi F., Casini G., Sellari-Franceschini S., Nardi M., Vitti P., Marcocci C., Marinò M. (2018). Antioxidant effects of beta-carotene, but not of retinol and vitamin E, in orbital fibroblasts from patients with Graves’ orbitopathy (GO). J. Endocrinol. Investig..

[103] Beltowski J. (2005). Statins and modulation of oxidative stress. Toxicol. Mech. Methods..

[104] Stein J.D., Childers D., Gupta S., Talwar N., Nan B., Lee B.J., Smith T.J., Douglas R. (2015). Risk factors for developing thyroid-associated ophthalmopathy among individuals with Graves disease. JAMA Ophthalmol..

[105] Nilsson A., Tsoumani K., Planck T. (2021). Statins decrease the risk of orbitopathy in newly diagnosed patients with Graves disease. J. Clin. Endocrinol. Metab..

[106] Lanzolla G., Sabini E., Leo M., Menconi F., Rocchi R., Sframeli A., Piaggi P., Nardi M., Marcocci C., Marinò M. (2021). Statins for Graves’ orbitopathy (STAGO): A phase 2, open-label, adaptive, single centre, randomised clinical trial. Lancet. Diabetes Endocrinol..

[107] Lanzolla G., Vannucchi G., Ionni I., Campi I., Sileo F., Lazzaroni E., Marinò M. (2020). Cholesterol Serum Levels and Use of Statins in Graves’ Orbitopathy: A New Starting Point for the Therapy. Front. Endocrinol..

[108] Bartalena L., Kahaly G.J., Baldeschi L., Dayan C.M., Eckstein A., Marcocci C., Marino M., Vaidya B., Wiersinga W.M. (2021). The 2021 European Group on Graves’ orbitopathy (EUGOGO) clinical practice guidelines for the medical management of Graves’ orbitopathy. Eur. J. Endocrinol..

[109] Yu W.K., Hwang W.L., Wang Y.C., Tsai C.C., Wei Y.H. (2021). Curcumin Suppresses TGF-β1-Induced Myofibroblast Differentiation and Attenuates Angiogenic Activity of Orbital Fibroblasts. Int. J. Mol. Sci..

[110] Shehzad A., Qureshi M., Anwar M.N., Lee Y.S. (2017). Multifunctional Curcumin Mediate Multitherapeutic Effects. J. Food Sci..

